# Association of viral loads of influenza A (H3N2) with age and care setting on presentation—a prospective study during the 2022-2023 influenza season in Spain

**DOI:** 10.1016/j.ijid.2024.107034

**Published:** 2024-06

**Authors:** Iván Sanz-Muñoz, Javier Sánchez-Martínez, Carla Rodríguez-Crespo, Irene Arroyo-Hernantes, Marta Domínguez-Gil, Silvia Rojo-Rello, Marta Hernández, José M Eiros

**Affiliations:** 1 National Influenza Centre, Valladolid, Spain; 2 Instituto de Estudios de Ciencias de la Salud de Castilla y León, ICSCYL, Soria, Spain; 3 Centro de Investigación Biomédica en Red de Enfermedades Infecciosas (CIBERINFECC), Madrid, Spain; 4 BioCritic, Group of Biomedical Research in Critical Medicine, Hospital Clínico Universitario de Valladolid, Valladolid, Spain; 5 Microbiology Unit, Hospital Universitario Río Hortega, Valladolid, Spain; 6 Microbiology Unit, Hospital Clínico Universitario de Valladolid, Valladolid, Spain; 7 Area of Microbiology, Faculty of Medicine, Universidad de Valladolid, Valladolid, Spain

**Keywords:** Influenza, Viral load, Cycle threshold, Sentinel, Hospitalized, Primary care

## Abstract

**Objectives:**

The aim of this study was to analyze the viral load (VL) using cycle threshold (Ct) in patients infected with influenza A (H3N2).

**Methods:**

This prospective study was conducted during the 2022-2023 influenza season in sentinel, non-sentinel, and hospitalized patients of Castilla y León (Spain). Respiratory samples were obtained from nasopharyngeal swabs and analyzed by quantitative reverse transcription-polymerase chain reaction specific for influenza A (H3N2) to obtain the Ct value.

**Results:**

A total of 1047 individuals were enrolled (174 [16.6%] sentinel, 200 [19.1%] non-sentinel, 673 [64.3%] hospitalized). The mean Ct value was lower in infants, young children, and in the elderly, with a sharp increase in the last from 65 years until 90 years. In addition, the lower Ct values were observed in non-sentinel patients and then in hospitalized patients, probably because non-sentinel are outpatients in the acute phase of the influenza infection.

**Conclusions:**

A higher VL (lower Ct value) is related to the extreme ages of life: children and the elderly. Furthermore, a higher VL is related with the care setting, being probably higher in outpatients because they are in the acute phase of the disease and slightly lower in hospitalized patients because they are attended during the post-acute phase.

## Highlights

• This is a prospective analysis of 1047 patients with A (H3N2) laboratory-confirmed infection.
• The analysis of the cycle threshold value by quantitative reverse transcription-polymerase chain reaction was used as a marker of viral load.
• Infants, children, and the elderly present the highest viral load.
• Outpatients showed higher viral loads than hospitalized patients.
• Age and care setting are important for viral load evaluation.

## Introduction

Because the precise significance of viral load (VL) test for the management and treatment of patients with respiratory viruses remains unknown, it is not a commonly used tool. However, since the COVID-19 pandemic, VL evaluation has gained popularity in the scientific community and health care providers owing to the possible application in daily clinical practice and research [1], [2], [3]. During the worst part of the pandemic when hospitals were overcrowded and a huge need for resources was the most important issue, the cycle threshold (Ct) value, a subrogate of the VL, was used as a criterion for discharging patients. In this regard, real-time quantitative reverse transcription-polymerase chain reaction (qRT-PCR) analysis is the easiest way to determine the Ct, keeping in mind that the lower the Ct, the higher the VL. In this way, it can be determined whether a patient has a higher or lower VL by having a precise value that is relatively available [4,5].

Despite that, there are many gaps of knowledge regarding the usefulness of the evaluation of VL in infectious respiratory pathology. A few works studying the respiratory syncytial virus and human metapneumovirus demonstrate the potential utility of VL measurement as a marker of the patient's prognosis, disease severity, and mortality risk [6], [7], [8], [9]. For both viruses, the severity of disease presentation in children and young adults correlates well with the VL.

Nevertheless, in the case of influenza viruses, it is not clear at all. A systematic review found that the 33% of the studies conducted for influenza determined that a higher VL was associated with mortality, increased disease severity/duration, intensive care unit (ICU) admission, and higher length of hospital stay [10]. Another study demonstrates that a high VL was a risk factor associated with a fatal outcome [11]. However, some other works did not find any relationship between VL and severity. For example, a work conducted during the 2009 A(H1N1)pdm09 pandemic showed that VL was similar in people experiencing severe and mild disease and no correlation was stablished with the duration of the illness [12]. Another study conducted in 2016 also showed no correlation of VL with severity but shows a direct correlation with body temperature [13].

Other works found interesting differences related to the type/subtype of influenza virus identified [14]. These authors determine that there are no differences between the samples collected in primary care/emergency units compared with those collected in hospitalization/ICU units for the A(H3N2) subtype; however, they found a higher VL for influenza B cases in patients who need hospitalization or ICU admittance. In contrast, other works showed no VL differences between type/subtypes [15]; however, the authors found a higher VL in patients who died of acute respiratory distress syndrome than those who survived. These data show that VL measurement could be useful for some specific groups, such as patients with acute respiratory distress syndrome.

There are other features of influenza VL that seems to be clearer. For instance, many works show that VL is greater in children and the elderly, probably because of the lower capacity of the immune system to fight infection compared with adults [16], [17], [18]. Other longitudinal works show that viral clearance is faster in outpatients than in hospitalized patients and young adults aged 15 to 49 years and that the resolution of some symptoms, such as cough and headache, is correlated with a decrease in VL [19]. Others correlate abnormal findings in chest X-ray and analytical parameters with a higher VL [20].

All these data show that there are many gaps to be addressed in the study of influenza VL measurement; therefore, more research is needed to further investigate the usefulness of the use of VL to characterize the features of influenza cases. Currently, there is a lack of local studies analyzing large cohorts of patients from different care settings. In this study, we aimed to analyze whether there are differences in VL based on the age, sex, and care setting of three Spanish cohorts of patients who tested positive for influenza treated in non-sentinel health care centers, in a sentinel network, and in hospitals.

## Materials and methods

### Study design and patient recruitment

Patients with acute respiratory infection (ARI) and severe ARI (SARI) who were diagnosed with laboratory-confirmed influenza in 2022-2023 were included in our prospective observational study. Samples were collected through the ARI and SARI surveillance systems of Castilla y León (Spain), and patients were recruited through three distinct health care settings. The first was through the Sentinel Surveillance Network of Castilla y León (Red VIGIRA, Vigilancia de las IRAs en Castilla y León) [21], which collects respiratory samples from patients with ARI using the VIGIRA program through primary care facilities set aside for this purpose. The system gathers samples from the first two patients (regardless of age and ARI characteristics) who visit the sentinel physician in each epidemiological week who fulfill the criteria for ARI [22]: sudden onset of illness accompanied by at least one symptom, such as cough, sore throat, dyspnea, and/or rhinitis. Health centers outside of the VIGIRA network served as the second site of care. They collected samples based on the attending physician's clinical criteria without setting a fixed quota using the same ARI case definition. Third, all hospitals in the region of Castilla y León that provided samples from patients with SARI were included using the following case definition [22]: the same as ARI plus a temperature rise above 38°C. In accordance with the criteria, all samples were taken 5 days after the onset of symptoms; however, the precise day of onset is unknown. The subjects provided written informed consent. The East-Valladolid Health Area's ethics committee approved this study, which was carried out in accordance with the Declaration of Helsinki and was assigned the code PI 21-2314.

### Methods

The National Influenza Center in Valladolid, Spain carried out specific influenza tests on the samples. Owing to the features of the Spanish epidemic of 2022-2023, it was decided to limit the analysis to subtype A (H3N2), the predominant virus of the epidemic. Using a monoplex qRT-PCR specific for subtype A (H3N2) and the CDC Influenza Virus Real-Time RT-PCR Panel, Influenza A (H3/H1 pdm09) Subtyping Panel V3), the virological analysis was performed by molecular diagnostics [23]. Earlier, Nextractor NX-48S automated extractor (Genolution, South Korea) and Savygen S Respiratory Extraction Kit reagents (Savyon Diagnostics, Israel), used previously for other quantitation protocols for SARS-CoV-2 [24], were used to extract the samples’ genetic material. The A (H3N2)-specific primers and probe are included in this reagent kit. Reagents for qRT-PCR, SuperScript III Platinum One-Step (Invitrogen, Waltham, MA, USA), were used. The polymerase chain reaction (PCR) cycling conditions as indicated by the Centers for Disease Control and Prevention were the following: 45 cycles of 95°C for 15 seconds and 55°C for 30 seconds, followed by 50°C for 30 minutes and 95°C for 15 minutes. Fluorescence acquisition was performed at the 55°C stage. A Roche L480 thermocycler (Roche, Basel, Switzerland) was used to conduct the PCR. After obtaining each sample's Ct data, it was added to a database for their analysis. A standard curve was not used because only the Ct value was used as a subrogate of VL.

### Statistical analysis

The statistical analysis was performed following two different approaches. First, the data were subjected to a descriptive analysis, which examined the variations in Ct values between age groups, sexes, and care settings. The age-based analysis was performed using a segregation using the classical age groups used in influenza studies. The Ct value between age groups and patient care settings was compared using a one-way analysis of variance with the Bonferroni test. The Student's *t* test was used to compare the Ct values between the two sexes. The statistical program GraphPad was used. Second, it was conducted a cubic spline model analysis using age as a continuous variable, and care setting as a factor was integrated in this analysis. The analysis was performed using a semi-parametric generalized additive model, which allows us modeling non-linear relationships between predictor variables and a response variable. The theoretical formula of the generalized additive model was formulated as follows:

$$g\left(E_{Y}\left(y│x\right)\right)=\beta_{0}+f_{1}\left(x_{1}\right)+f_{2}\left(x_{2}\right)\ldots +{f_{p}}^{\left(xp\right)}$$

The package of this function in R software, version 4.3.2., was “mgcv”. A *P*-value <0.05 was considered statistically significant.

## Results

### Features of patients

A total of 1047 subjects were recruited, of whom 174 (16.6%) were sentinel, 200 (19.1%) were non-sentinel, and 673 (64.3%) were hospitalized. The mean overall age was 34.9 (confidence interval [CI] 95%, 33.1-36.8) years. The distribution of the individual age groups by health care facility is described in Table 1.

**Table 1:** Demographic characteristics (sex and age) of the patients included in the study and place of health care (sentinel, non-sentinel, and hospitalized patients).

**Input**		Total	Sentinel	Non-sentinel	Hospitalized
		**N (%)**	**N (%)**	**N (%)**	**N (%)**
Total		1,047 (100)	174 (16.6)	200 (19.1)	673 (64.3)
Age	Mean age (CI 95%)				
0-4 yrs	2.5 (2.4-2.7)	202 (19.3)	46 (22.8)	42 (20.8)	114 (56.4)
5-14 yrs	8.4 (7.9-8.9)	140 (13.4)	41 (29.3)	23 (16.4)	76 (54.3)
15-39 yrs	25.3 (24-5-26.0)	326 (31.1)	44 (13.5)	63 (19.3)	219 (67.2)
40-65 yrs	52.6 (51.-53.8)	143 (13.7)	28 (19.6)	27 (18.9)	88 (61.5)
65-79 yrs	72.9 (72.2-73.6)	120 (11.5)	14 (11.7)	20 (16.7)	86 (71.6)
>80 yrs	89.3 (88.2-90.3)	116 (11.0)	1 (0.9)	25 (21.6)	90 (77.5)
Sex					
Male	NA	540 (51.6)	97 (18.0)	105 (19.4)	338 (62.6)
Female	NA	507 (48.4)	95 (18.7)	77 (15.2)	335 (66.1)

CI 95%, confidence interval 95%; NA, non-applicant; yrs, years.

### Analysis of the cycle threshold values by age and care setting

First, a global analysis of variations in Ct values by age group, sex, and patient care location was performed. There were some differences between Ct values when those values were compared between age groups. For instance, the mean Ct value was significantly lower in children aged 0-4 years than in adults aged 40-64 years (*P* <0.05). There were also notable variations in the Ct values between patient care settings. These Ct values were significantly higher in sentinel patients than in non-sentinel (*P* <0.01) and hospitalized (*P* <0.05) patients. Because there were no discernible differences in the mean Ct value between the sexes, we decided not to conduct further investigation.

### Modelization of cycle threshold values by the age and the care setting patients

**Figure 1:**
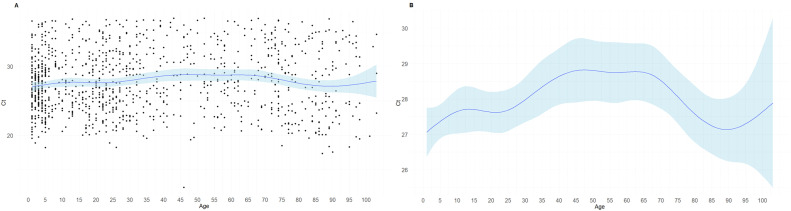
Results of the spline model for age-based Ct value changes. Data are presented in dots. Panel A shows the mean of predictions (solid line) and a 95% interval (shaded area) of viral load by age. Panel B shows the same prediction and 95% interval eliminating the dots to increase the visualization of differences. Ct, cycle threshold.

Because the descriptive analysis did not explain enough the differences between age and care settings, we used the cubic spline model to study the differences in age features using the age as a continuous variable. The spline model was significant (*P* <0.0001) but the adjusted R^2^ was very low (R^2^_adj_ = 0.0133). This model showed that the lower Ct values were observed in infants, young children, and in the elderly and the highest values were detected in the adults (Figure 1). In fact, in the elderly, we observed a sharp decline in the Ct values from 65 until 90 years old.

**Figure 2:**
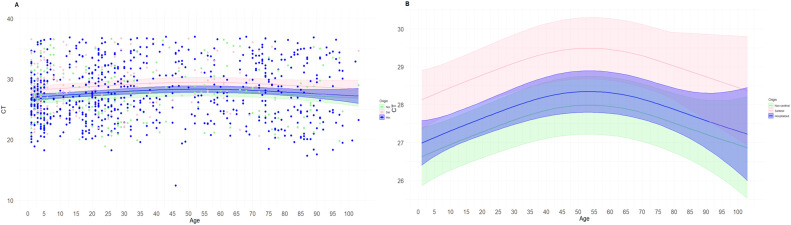
Results of the spline model for age-based Ct value changes organized by care setting. Data are presented in dots. Panel A shows the mean of predictions (solid line) and a 95% interval (shaded area) of viral load by age. Panel B shows the same prediction and 95% interval eliminating the dots to increase the visualization of differences. Ct, cycle threshold.

After that, we analyzed the dynamic of the Ct values based on age and care setting at the same time (Figure 2). The spline model showed significant differences among non-sentinel and sentinel patients (*P* <0.01) and between hospitalized and sentinel patients (*P* <0.01). In contrast, there are no significant differences between non-sentinel and hospitalized (*P* >0.05) patients. The adjusted R^2^ was very low (R^2^_adj_ = 0.019). Lower Ct values were observed in patients attended in non-sentinel primary care, followed by hospitalized patients, and the last who showed the higher Ct values were patients attended by the sentinel network. After this modelization, we made a table (Table 2) predicting the Ct values and 95% CI of each year of age based on the care setting where the patient was attended.

**Table 2:** Ct mean values predicted by the cubic spline model and 95% CI based on each age of life and the care setting where the treatment of the patient took place.

Age	Non-sentinel			Hospitalized			Sentinel		
	Predicted Ct	Low CI 95%	High CI 95%	Predicted Ct	Low CI 95%	High CI 95%	Predicted Ct	Low CI 95%	High CI 95%
1	26.6	25.9	27.4	27.0	26.4	27.6	28.1	27.4	28.9
2	26.7	25.9	27.4	27.0	26.5	27.6	28.2	27.4	28.9
3	26.7	26.0	27.4	27.1	26.5	27.6	28.2	27.5	28.9
4	26.7	26.0	27.5	27.1	26.6	27.6	28.2	27.5	29.0
5	26.8	26.1	27.5	27.1	26.6	27.6	28.3	27.6	29.0
6	26.8	26.1	27.5	27.2	26.7	27.7	28.3	27.6	29.0
7	26.8	26.2	27.5	27.2	26.7	27.7	28.3	27.6	29.1
8	26.9	26.2	27.6	27.2	26.8	27.7	28.4	27.7	29.1
9	26.9	26.2	27.6	27.3	26.8	27.7	28.4	27.7	29.1
10	26.9	26.3	27.6	27.3	26.9	27.8	28.5	27.8	29.1
11	27.0	26.3	27.7	27.3	26.9	27.8	28.5	27.8	29.2
12	27.0	26.3	27.7	27.4	26.9	27.8	28.5	27.8	29.2
13	27.1	26.4	27.7	27.4	27.0	27.8	28.6	27.9	29.3
14	27.1	26.4	27.8	27.4	27.0	27.9	28.6	27.9	29.3
15	27.1	26.4	27.8	27.5	27.0	27.9	28.6	27.9	29.3
16	27.2	26.5	27.8	27.5	27.1	27.9	28.7	28.0	29.4
17	27.2	26.5	27.9	27.5	27.1	28.0	28.7	28.0	29.4
18	27.2	26.5	27.9	27.6	27.1	28.0	28.7	28.0	29.4
19	27.2	26.6	27.9	27.6	27.2	28.0	28.8	28.0	29.5
20	27.3	26.6	28.0	27.6	27.2	28.1	28.8	28.1	29.5
21	27.3	26.6	28.0	27.7	27.2	28.1	28.8	28.1	29.5
22	27.3	26.7	28.0	27.7	27.3	28.2	28.8	28.1	29.6
23	27.4	26.7	28.1	27.7	27.3	28.2	28.9	28.2	29.6
24	27.4	26.7	28.1	27.8	27.3	28.2	28.9	28.2	29.6
25	27.4	26.7	28.1	27.8	27.3	28.3	28.9	28.2	29.7
26	27.5	26.8	28.2	27.8	27.4	28.3	29.0	28.2	29.7
27	27.5	26.8	28.2	27.9	27.4	28.3	29.0	28.3	29.7
28	27.5	26.8	28.2	27.9	27.4	28.4	29.0	28.3	29.8
29	27.6	26.9	28.3	27.9	27.5	28.4	29.1	28.3	29.8
30	27.6	26.9	28.3	28.0	27.5	28.4	29.1	28.3	29.8
31	27.6	26.9	28.3	28.0	27.5	28.5	29.1	28.4	29.9
32	27.7	26.9	28.4	28.0	27.5	28.5	29.2	28.4	29.9
33	27.7	27.0	28.4	28.0	27.6	28.5	29.2	28.4	29.9
34	27.7	27.0	28.4	28.1	27.6	28.6	29.2	28.4	30.0
35	27.7	27.0	28.5	28.1	27.6	28.6	29.2	28.5	30.0
36	27.8	27.0	28.5	28.1	27.6	28.6	29.3	28.5	30.0
37	27.8	27.1	28.5	28.1	27.6	28.7	29.3	28.5	30.1
38	27.8	27.1	28.5	28.2	27.7	28.7	29.3	28.5	30.1
39	27.8	27.1	28.6	28.2	27.7	28.7	29.3	28.6	30.1
40	27.9	27.1	28.6	28.2	27.7	28.7	29.4	28.6	30.1
41	27.9	27.1	28.6	28.2	27.7	28.8	29.4	28.6	30.2
42	27.9	27.1	28.6	28.3	27.7	28.8	29.4	28.6	30.2
43	27.9	27.2	28.7	28.3	27.7	28.8	29.4	28.6	30.2
44	27.9	27.2	28.7	28.3	27.7	28.8	29.4	28.6	30.2
45	27.9	27.2	28.7	28.3	27.8	28.8	29.4	28.6	30.2
46	27.9	27.2	28.7	28.3	27.8	28.8	29.4	28.6	30.2
47	28.0	27.2	28.7	28.3	27.8	28.9	29.5	28.7	30.3
48	28.0	27.2	28.7	28.3	27.8	28.9	29.5	28.7	30.3
49	28.0	27.2	28.7	28.3	27.8	28.9	29.5	28.7	30.3
50	28.0	27.2	28.7	28.3	27.8	28.9	29.5	28.7	30.3
51	28.0	27.2	28.7	28.3	27.8	28.9	29.5	28.7	30.3
52	28.0	27.2	28.8	28.3	27.8	28.9	29.5	28.7	30.3
53	28.0	27.2	28.8	28.3	27.8	28.9	29.5	28.7	30.3
54	28.0	27.2	28.8	28.3	27.8	28.9	29.5	28.7	30.3
55	28.0	27.2	28.8	28.3	27.8	28.9	29.5	28.7	30.3
56	28.0	27.2	28.7	28.3	27.8	28.9	29.5	28.7	30.3
57	28.0	27.2	28.7	28.3	27.8	28.9	29.5	28.7	30.3
58	28.0	27.2	28.7	28.3	27.8	28.9	29.5	28.7	30.3
59	28.0	27.2	28.7	28.3	27.8	28.9	29.5	28.7	30.3
60	28.0	27.2	28.7	28.3	27.8	28.9	29.5	28.6	30.3
61	27.9	27.2	28.7	28.3	27.8	28.9	29.4	28.6	30.3
62	27.9	27.2	28.7	28.3	27.8	28.8	29.4	28.6	30.3
63	27.9	27.2	28.7	28.3	27.7	28.8	29.4	28.6	30.2
64	27.9	27.1	28.7	28.3	27.7	28.8	29.4	28.6	30.2
65	27.9	27.1	28.7	28.3	27.7	28.8	29.4	28.6	30.2
66	27.9	27.1	28.6	28.2	27.7	28.8	29.4	28.6	30.2
67	27.9	27.1	28.6	28.2	27.7	28.8	29.4	28.5	30.2
68	27.8	27.1	28.6	28.2	27.7	28.7	29.3	28.5	30.2
69	27.8	27.1	28.6	28.2	27.6	28.7	29.3	28.5	30.1
70	27.8	27.0	28.5	28.2	27.6	28.7	29.3	28.5	30.1
71	27.8	27.0	28.5	28.1	27.6	28.7	29.3	28.4	30.1
72	27.7	27.0	28.5	28.1	27.6	28.6	29.2	28.4	30.1
73	27.7	27.0	28.5	28.1	27.5	28.6	29.2	28.4	30.1
74	27.7	26.9	28.4	28.0	27.5	28.6	29.2	28.4	30.0
75	27.7	26.9	28.4	28.0	27.5	28.6	29.2	28.3	30.0
76	27.6	26.9	28.4	28.0	27.5	28.5	29.1	28.3	30.0
77	27.6	26.8	28.4	28.0	27.4	28.5	29.1	28.3	30.0
78	27.6	26.8	28.3	27.9	27.4	28.5	29.1	28.2	29.9
79	27.5	26.8	28.3	27.9	27.4	28.5	29.0	28.2	29.9
80	27.5	26.7	28.3	27.9	27.3	28.4	29.0	28.2	29.9
81	27.5	26.7	28.3	27.8	27.3	28.4	29.0	28.1	29.9
82	27.5	26.7	28.2	27.8	27.2	28.4	29.0	28.1	29.8
83	27.4	26.6	28.2	27.8	27.2	28.4	28.9	28.0	29.8
84	27.4	26.6	28.2	27.8	27.2	28.4	28.9	28.0	29.8
85	27.4	26.5	28.2	27.7	27.1	28.3	28.9	28.0	29.8
86	27.3	26.5	28.2	27.7	27.1	28.3	28.8	27.9	29.8
87	27.3	26.5	28.2	27.7	27.0	28.3	28.8	27.9	29.8
88	27.3	26.4	28.1	27.6	27.0	28.3	28.8	27.8	29.7
89	27.3	26.4	28.1	27.6	26.9	28.3	28.8	27.8	29.7
90	27.2	26.3	28.1	27.6	26.9	28.3	28.7	27.7	29.7
91	27.2	26.3	28.1	27.6	26.8	28.3	28.7	27.7	29.7
92	27.2	26.2	28.1	27.5	26.7	28.3	28.7	27.6	29.7
93	27.1	26.2	28.1	27.5	26.7	28.3	28.6	27.6	29.7
94	27.1	26.1	28.1	27.5	26.6	28.3	28.6	27.5	29.7
95	27.1	26.1	28.1	27.4	26.6	28.3	28.6	27.5	29.7
96	27.1	26.0	28.1	27.4	26.5	28.3	28.6	27.4	29.7
97	27.0	25.9	28.1	27.4	26.4	28.3	28.5	27.3	29.7
98	27.0	25.9	28.1	27.4	26.4	28.4	28.5	27.3	29.7
99	27.0	25.8	28.1	27.3	26.3	28.4	28.5	27.2	29.7
100	26.9	25.7	28.2	27.3	26.2	28.4	28.4	27.1	29.8
101	26.9	25.7	28.2	27.3	26.1	28.4	28.4	27.1	29.8
102	26.9	25.6	28.2	27.3	26.1	28.4	28.4	27.0	29.8
103	26.9	25.5	28.2	27.2	26.0	28.5	28.4	26.9	29.8

CI, confidence interval; Ct, cycle threshold.

## Discussion

To the best of our knowledge, to date, our work is one of the largest studies analyzing influenza VL in different health care settings. Our data showed a clear relationship between VL and age. Although the robustness of the spline model used was low, probably owing to the great dispersion of the Ct values among the ages, these data clearly show that greater VL (lower Ct value) was detected in infants and in the elderly and was the lowest in adults, describing an inverted “U” shape.

These data are in line with other studies that found that the highest VL was also present in children and in the elderly [16], [17], [18], possibly because of their poorer ability to control the virus, making them their primary carriers [25], [26], [27] and the groups with the higher burden of disease [28]. However, we believe that these data may also show behavioral patterns whereby children and the elderly are brought by the caretakers to medical attention earlier than adults. Because we do not have data on symptoms and severity of infection, we cannot specify which of the two hypotheses is true or whether both are implicated in these results.

In our study, we also demonstrated that, although VL is higher in the elderly than in adults, it is not homogeneous within this group. One of the most interesting results of our study is that we have been able to discover that the rise of the influenza VL started at the age of 65 years and is described as a sharp increase until the age of 90 years. With these data, we have shown that influenza infection in a person aged 65 years does not the same behavior as in a person aged 90 years because the higher VL in the latter could be related to the greater severity of infection in these patients because of their biological fragility [29] and their immunosenescence [30], [31], [32], [33].

In the case of care setting differences, although the model used did not have a great robustness, we saw a clear tendency in the data indicating that there are some interesting differences between care settings. Our data showed that patients attended in non-sentinel health centers had the highest VL, followed by those in hospitals, and, finally, those attended at the sentinel network. It is surprising that there are noticeable differences between patients who are in a sentinel network and those who are not, given that primary care is provided for both groups. The sentinel network has very specific instructions for sample collection, which mandate that the first two patients of the week must be sampled, regardless of the patient's disease severity. However, those primary care physicians who are not a part of a sentinel network will only take samples in the most severe cases for a better management of their patients.

According to our findings, samples collected in primary care settings that do not pertain to a sentinel network represent people with acute disease symptoms, probably with the highest VL because they are in the acute phase of the disease within the first 1-2 days after infection [19,34]. In contrast, patients in the sentinel network promote a randomized selection that can represent all spectra of the disease but not exactly the ones that have a more severe disease course. After that, people with severe disease who require hospitalization present a lower VL than non-sentinel, probably because they are in the post-acute phase of the infection [35].

We believe that this reflects that VL is not only related to the severity of symptoms [20,36] but also to the infectious stage of the virus at the time of sampling [34,37]. However, many studies do not emphasize the possible differences between hospitalized patients and those treated in health centers, which would show different stages of the same disease. Our study provides novel data, which, although could not be contrasted with the patient's symptomatology nor with the kinetics of the virus, seem to indicate indirectly that VL is related to severity and viral kinetics, supporting other previously published studies. This behavior was observed for all ages, demonstrating that, despite this, the severity and the infectious phase of the virus are directly related to the VL. In addition, we used a methodology that can be used for prediction of the Ct value based on age and care setting.

The higher VL found in children and the elderly has clear implications in public health and patient management. First, our data, together with those of other studies, demonstrate the clear role of late life in the burden of disease and the spread of influenza. This is important not only in the community but also when the patient requires hospitalization because they can be a source of nosocomial infection. Second, the results of our study show that VL is higher in patients who are managed in non-sentinel primary care, followed by those in hospital settings, which may guide the understanding of the dynamics of viral kinetics and encourage the viral diagnostic in primary care, which could benefit the patient for a better treatment and management.

The main limitation of our study is that without the data of the symptoms of the patients included, we cannot ascertain whether the differences found in the VL are related to a higher or lower severity of influenza infection. Moreover, it is uncertain whether the clinical characteristics of the study participants, such as their comorbidities, immunosuppressive conditions, or other conditions that can affect viral replication, are implicated in this variation of VL. In addition, we did not use any standard during the PCR protocols to ascertain the exact copy number of the VL, using only the Ct value. However, we believe that with the aim of the work, this is does not limit the conclusions obtained.

## Conclusion

The highest influenza VL values were observed at the most extreme ages of life: children and the elderly. Moreover, the VL increased gradually from 65 to 90 years of age, demonstrating that it is dependent on the age of the individual. In contrast, the highest VL was found in primary care patients not included in the sentinel network, followed by hospitalized patients. This shows that VL is influenced by two different factors: the viral phase at the time of sampling and the severity of symptoms. Therefore, VL measurement can be used as an indirect indicator of both factors. However, further research is needed to clarify the exact VL values that could help in clinical management.

## CRediT authorship contribution statement

**Iván Sanz-Muñoz:** Conceptualization, Methodology, Investigation, Formal analysis, Resources, Supervision, Visualization, Writing – original draft, Writing – review & editing. **Javier Sánchez-Martínez:** Methodology, Investigation, Supervision. **Carla Rodríguez-Crespo:** Methodology, Investigation. **Irene Arroyo-Hernantes:** Methodology, Formal analysis, Writing – original draft. **Marta Domínguez-Gil:** Formal analysis, Investigation, Writing – original draft, Writing – review & editing. **Silvia Rojo-Rello:** Formal analysis, Investigation, Writing – original draft, Writing – review & editing. **Marta Hernández:** Writing – review & editing. **José M Eiros:** Conceptualization, Formal analysis, Methodology, Investigation, Resources, Supervision, Visualization, Writing – original draft, Writing – review & editing.

## Declarations of competing interest

The authors have no competing interests to declare.

## Appendix Supplementary materials

Image, application 1
