# Supplementary material for: Association of viral loads of influenza A (H3N2) with age and care setting on presentation—a prospective study during the 2022-2023 influenza season in Spain
Source: Int J Infect Dis. 2024 Jun;143:None. doi: 10.1016/j.ijid.2024.107034 (PMC11068591; doi:10.1016/j.ijid.2024.107034)
Supplement: Supplementary file 1 [file mmc1.pdf]

International Journal of Infectious Diseases
AUTHORSHIP AGREEMENT

For all submissions to International Journal of Infectious Diseases (THEIJID), request of authorship change during review process should be made to the Editorial Office of THEIJID. The form below should be completed for any authorship changes including adding new authors, removing existing authors, reordering existing authors, or adjusting equal contributor status of existing authors. Corresponding author should confirm that all authors meet the criteria for authorship as outlined by Committee on Publication Ethics (COPE) and that all authors agree to the change. All authors who have been added, removed, or reordered need to confirm that they agree to the change by signing the form. Please fill and return this form in time. The concerned submission will be put on hold for further processing until the editorial office receives the completed form.

Manuscript number: THEIJID-D-24-00136R1

Manuscript title: Association of viral loads of influenza A(H3N2) with age and care setting on presentation – a prospective study during the 2022/23 influenza season in Spain

Would you like to change the authorship of your paper? (Please tick one of the following):

- No (No signature is needed if selected)
- **Yes** (please fill in box below with all necessary signatures):

The nature of change(s) in authorship (Please check one of the following):

- Change de Corresponding Author:
- **Add new author(s);**
- Remove existing author(s);
- Change the order of authorship;
- Others (please specify):

Detailed reason for the change

We had had to include one more author (Ms. Irene Arroyo-Hernantes) because we had had to perform deeper statistical analysis due to the petition of the referee 2. This new author had performed the statistical protocols, described the data and performed the new figures suggested by referee 2. For that reason, we consider essential to incorporate her as an author. Because of that, we included in the authors contribution section what she has done in the manuscript. I hope this can clarify her incorporation. I confirm that all authors agree with including this new author, and they will sign below this document.

Indicate the specific change:

Adding a new author

**Name**	Irene Arroyo-Hernantes
**Email address**	iarroyoh@saludcastillayleon.es
**Institution**	BioCritic, Group of Biomedical Research in Critical Medicine, Hospital Clínico Universitario de Valladolid, Spain
**Specific contribution**	New statistical analysis suggested by the referee 2. She has deep knowledge on the spline models suggested so she analyzed the data with the new insight and performed the new figures and tables.

Removing an existing author

Name	
Email address	
Institution	
Agree to be acknowledged	

Complete author order AFTER change (please note any authors with equal contribution as first authors or corresponding authors)

Order	Author name BEFORE change	Author name AFTER change	Signature
1	Iván Sanz-Muñoz	Iván Sanz-Muñoz	[illegible]
2	JAVIER SÁNCHEZ-MARTÍNEZ	JAVIER SÁNCHEZ-MARTÍNEZ	[illegible]
3	CARLA RODRÍGUEZ-CRESPO	CARLA RODRÍGUEZ-CRESPO	[illegible]
4	MARTA DOMÍNGUEZ-GIL	IRENE ARROYO-HERNANDO	[illegible]
5	Silvia Rojo-Rello	MARTA DOMÍNGUEZ-GIL	[illegible]
6	MARTA HERNÁNDEZ	Silvia Rojo-Rello	[illegible]
7	José M. Eiros	MARTA HERNÁNDEZ	[illegible]
8		José M. Eiros	[illegible]
9			
10			

Approval of the final version of the manuscript to be submitted, all authors must sign the table above to indicate agreeing with the changed authorship (all authors must be listed, full name in print and signatures are needed from all).
